# Schistosoma “Eggs-Iting” the Host: Granuloma Formation and Egg Excretion

**DOI:** 10.3389/fimmu.2018.02492

**Published:** 2018-10-29

**Authors:** Christian Schwartz, Padraic G. Fallon

**Affiliations:** ^1^School of Medicine, Trinity Biomedical Sciences Institute, Trinity College Dublin, Dublin, Ireland; ^2^National Children's Research Centre, Our Lady's Children's Hospital, Dublin, Ireland; ^3^Trinity Translational Medicine Institute, St James's Hospital, Trinity College Dublin, Dublin, Ireland

**Keywords:** schistosoma, inflammation, granuloma, egg, excretion, regulation, intestine, liver

## Abstract

Schistosomiasis is a major cause of morbidity in humans invoked by chronic infection with parasitic trematodes of the genus Schistosoma. Schistosomes have a complex life-cycle involving infections of an aquatic snail intermediate host and a definitive mammalian host. In humans, adult male and female worms lie within the vasculature. Here, they propagate and eggs are laid. These eggs must then be released from the host to continue the life cycle. *Schistosoma mansoni* and *Schistosoma japonicum* reside in the mesenteric circulation of the intestines with egg excreted in the feces. In contrast, *S. haematobium* are present in the venus plexus of the bladder, expelling eggs in the urine. In an impressive case of exploitation of the host immune system, this process of Schistosome “eggs-iting” the host is immune dependent. In this article, we review the formation of the egg granuloma and explore how *S. mansoni* eggs laid in vasculature must usurp immunity to induce regulated inflammation, to facilitate extravasation through the intestinal wall and to be expelled in the feces. We highlight the roles of immune cell populations, stromal factors, and egg secretions in the process of egg excretion to provide a comprehensive overview of the current state of knowledge regarding a vastly unexplored mechanism.

## Introduction

Schistosomiasis (Bilharzia) is one of the worlds most common parasitic infections with over 200 million people requiring preventive treatment in 2016 (1, 2). While the majority of people at risk live in the endemic regions of Africa, *Schistosoma* species are also prevalent in the Middle East, the Caribbean, South America, and South East Asia. Autochthonous transmission of schistosomes has also been reported in Corsica, France (3, 4). Using novel, more sensitive diagnostic techniques to reveal “egg-negative/worm-positive schistosomiasis,” Colley et al. highlighted that the global prevalence of schistosomiasis may actually exceed current estimates (5). The main human pathogenic species causing intestinal schistosomiasis are *Schistosoma mansoni, Schistosoma japonicum, Schistosoma mekongi, Schistosoma intercalatum*, and *Schistosoma guineensis*. with *Schistosoma haematobium* causing urogenital schistosomiasis. While *S. haematobium* is a major cause of mortality, frequently causing renal failure, chronic morbidity is the major health concern with schistosome infection causing 3.3 million disability-adjusted life years (6). For the purpose of this review we will concentrate on the most prevalent species causing intestinal schistosomiasis, *S. mansoni*.

*S. mansoni* is well-adapted to chronically infect humans as a result of ~200,000 years of co-evolution with modern humans (7). This is reflected by the life-span of *S. mansoni* worms estimated to be 5.7–10.5 years in human hosts (8). Evidently, successful adaptation has established a host-parasite relation such that asymptomatic infection are present in more than 90% of individuals, however, some infected develop hepatic fibrosis, severe hepatosplenomegaly, and portal hypertension (9). Immunopathology during schistosome infection of humans is predominately caused by granulomatous inflammation around parasite eggs that are trapped in various organs. In this review, we will focus on *S. mansoni* and the immune-dependent process of egg granuloma formation, which facilitates the parasite egg excretion from the mammalian host and completetion of the trematodes life cycle.

## Life cycle of *schistosoma* SPP.

Schistosoma species have complex life-cycles involving infection of a freshwater snail intermediate host as well as a mammalian definitive host, such as humans. The *S. mansoni* egg stages are excreted from the human host within fecal material (or urine in case of *S. haematobium*). Under optimal conditions the eggs hatch in fresh water and–via asexual replication in the intermediate snail host, *Biomphalaria* genus for *S. mansoni*–thousands of free-swimming infective cercariae are released into the water. The cercariae locate a mammalian host and penetrate the skin, and then transform to the now so-called schistosomulae stage. The schistosomulae remain in the skin for several days, after which they enter the circulation via the lymphatics and venules to reach the lung 5–7 days after skin penetration. After >2 weeks, they re-enter the circulation and reach the hepatoportal circulation. Here, they remain and sexually mature into adult male or females worms after encountering a partner of the opposite sex. The monogamous pair migrate to the mesenteric veins, mate and begin egg production after ~28 days. Adult *S. mansoni* worms are predominantly found in the small inferior mesenteric blood vessels that surround the colon and caecum. Eggs laid by female worms are deposited onto the endothelial lining of the capillary walls. From here, the eggs are either disseminated through the blood flow into other organs or they translocate through the intestinal epithelia into the intestinal lumen. The eggs are metabolically active and highly antigenic–they evoke inflammation that leads to the formation of a granuloma around the egg necessary for the translocation through the lamina propria. Excretion of eggs within the fecal material then completes the parasites life cycle.

Acute clinical symptoms may include the development of a light rash, commonly referred to as “swimmers itch.” “Katayama fever” is characterized by fever, fatigue, and dry cough–among other symptoms–and may occur 2–12 weeks after infection resulting from a systemic reaction against the migrating schistosomulae. During chronic stages of infection, half to two thirds of the eggs deposited in mesenteric venules are swept away in the circulation to multiple organs, with the majority ending up in the liver (10). In the liver, granulomatous inflammation around eggs and the subsequent fibrosis lead to the major pathologies associated with schistosomiasis mansoni. Fibrosis in the liver portal tract often leads to obstructive portal lesions and portal hypertension, and may result in gastrointestinal bleeding, hepatic encephalopathy and liver failure. Interestingly, despite the constant translocation of eggs from the vasculature into the intestinal lumen, cases of *S. mansoni*-associated sepsis are rare, reinforcing the highly adapted process of egg excretion (11).

## Schistosome animal models are required to study the egg excretion process

Human studies are generally undertaken in endemic settings and commonly are based on observations before and after drug treatment for schistosomiasis. While analysis of human material (blood, tissue, stool) are definitely required to translate findings from animal experiments to the human system (12), these samples come with some caveats: heterogenic background, medical history, co-infections, environmental factors, etc. Perhaps these may be overcome someday by the growing computational power and the use of high throughput “omics.” More intriguingly, the ongoing studies involving controlled human infection with *S. mansoni* will provide new insight to most aspects of infection of humans (13). However, to formally address the egg excretion process in humans the deliberate experimental chronic infection with mixed sex cercariae, resulting in egg producing male and female worm infections and egg associated tissue immunopathology leading to morbidity and the risk of mortality, may pose ethical concerns. Alternatively, longitudinal studies in endemic areas are logistically demanding as they would require colonoscopy to access the intestinal epithelium.

Animal models have greatly advanced our understanding of the pathophysiology of schistosome infection. While chimpanzees and baboons are the most faithful models recapitulating all features of human schistosomiasis including peri-portal fibrosis and intestinal lesions (14–18), today the most frequently used species is the mouse, although not all findings are translatable. This suitability of mice as a model, must be considered in the context that *S. mansoni* may have adapted some 125,000 years ago to humans from the rodent trematode *S. rodhaini* (19). While the mouse shows differences in hepatic fibrosis and pathology, which is more associated with the granulomatous response to trapped parasite eggs in the liver and intestine (20), the development of granulomas adequately reflects human disease. As the process of egg excretion is dependent on the immune-dependent formation of granulomatous inflammation mice are a well-suited model to mechanistically study egg translocation, which is facilitated by the availability of reagents and transgenic mice ultimately guiding the studies on infected humans. In this review, we will rely mostly on data generated in mice as a model of *S. mansoni* infection and immunopathology.

## Differences in granulomatous inflammation of liver and intestine

### Immune response to *S. mansoni*

Following infection, schistosomulae migrate through the host body and a type 1 immune dominated response is elicited, persisting for ~5 weeks. This response is characterized by increased release of Interleukin (IL)-12 and interferon (IFN)-γ and is mainly targeted at worm antigens. However, as the parasite matures and starts to produce eggs (~5–6 weeks postinfection), a shift toward a type 2-biased immune response occurs. Consequently, IFN-γ production decreases, while CD4^+^ T helper (Th) 2 cell polarization is induced. The switch from type 1 to type 2 is elicited by the eggs released by adult female worms. Indeed, whole *S. mansoni* eggs or their soluble egg antigens (SEA) potently induce type 2 responses when injected into naïve mice (21). Importantly, a defect in switching the type of immune response leads to aberrant intestinal inflammation and fatal disease (22, 23). The protective type 2 immune response is characterized by expansion of Th2 cells, eosinophils, and basophils, increased production of IL-4, IL-5, and IL-13, an isotype switch toward IgG1 and IgE, as well as polarization of macrophages toward the M2 phenotype (24, 25). During the chronic phase of infection (>3 months), the magnitude of the Th2 response decreases, coincident with a reduction in granulomatous inflammation around eggs, and regulatory T and B cells emerge leading to a state of immune hyporesponsiveness.

Granuloma formation around eggs trapped within hepatic and intestinal tissue is a hallmark of schistosome infection and the major cause of pathology in infected hosts. However, the egg granuloma functions for both the host and parasite (26): (a) intestinal granulomatous inflammation facilitates the translocation process for the egg into the gastrointestinal lumen, (b) it ameliorates bacterial translocation from the intestine into the circulation of the host, (c) it protects host tissues from exaggerated immune responses against the antigenic eggs, and (d) it ultimately benefits the adult parasites to keep the host intact.

### Intestinal granulomas

**Figure 1:**
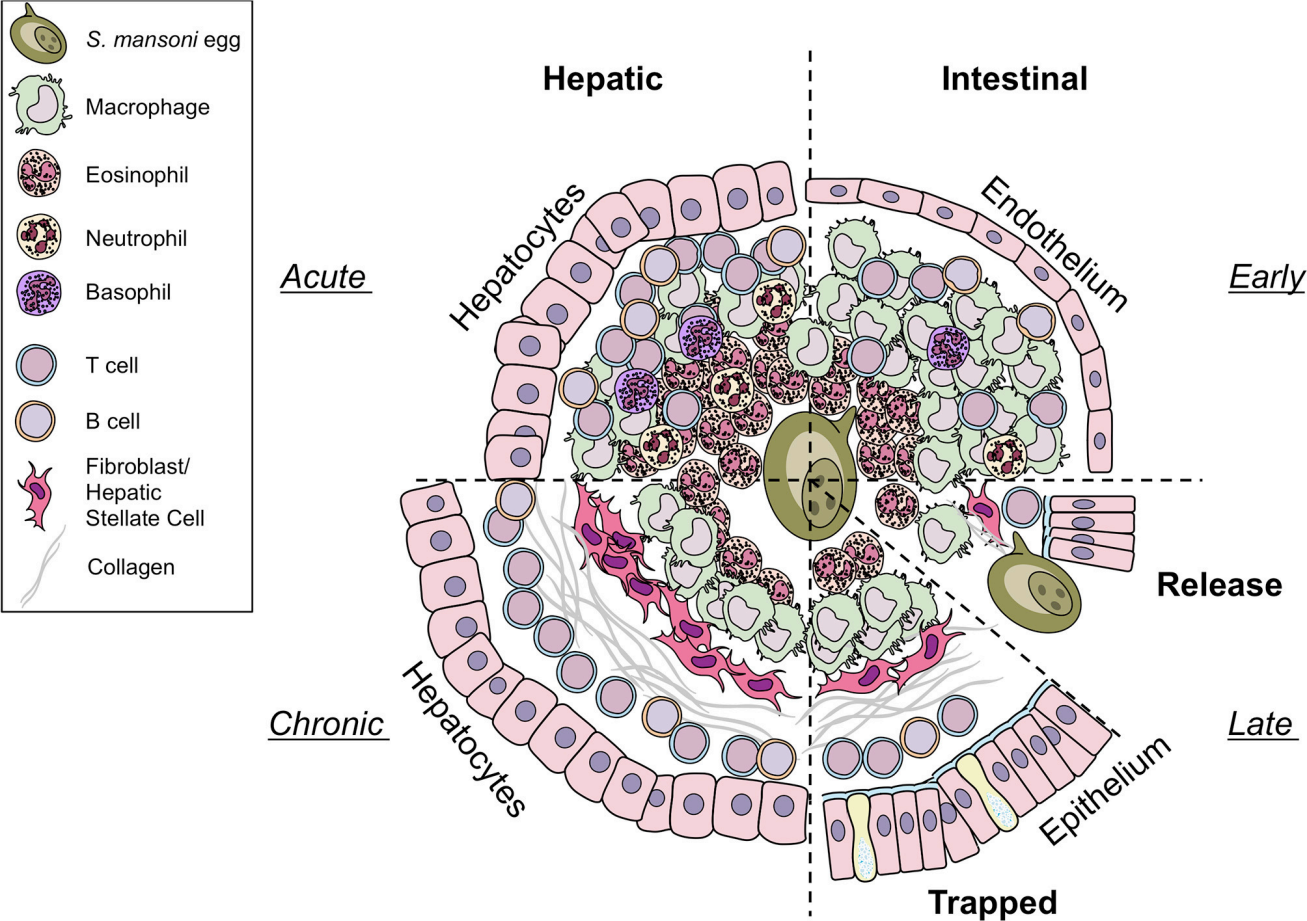
Differences in hepatic and intestinal granuloma composition. Cellular granuloma composition in the liver (left) and the intestine (right). While early (upper half) granulomas may appear similar, intestinal granulomas harbor less eosinophils, T cells, and B cells than hepatic granulomas, more macrophages are present. Only few neutrophils and basophils can be observed in both sites. During later stages (lower half) eggs in the liver become trapped and fibrosis develops. In contrast, eggs deposited in the gut must be released to the intestinal lumen by yet to define mechanisms. However, they may also become trapped and may resemble chronic liver granulomas in shape and collagen content. Illustrations modified from Servier Medical Art, licensed under a Creative Common Attribution 3.0 Unported License.

Female *S. mansoni* worms deposit their eggs close to the vasculature surrounding the intestines and exploit the host protective mechanism to facilitate egg transgression in order to get them transported through the endothelium and intestinal wall into the gut lumen. It has been estimated that *S. mansoni* eggs are viable for ~3 weeks indicating that eggs complete the transition through the intestinal wall well within that time frame but in no less than 6 days (27). Therefore, the intestinal granulomas will have characteristics of what is seen in early stage liver granulomas (Figure 1). Moreover, histological analysis has shown that colon granuloma composition differs from liver granulomas harboring more macrophages but less eosinophils, T and B cells (28). However, to date, a comprehensive in-depth characterization of the composition of the intestinal granuloma remains to be published. Due to the longevity of infection and constant egg deposition, assessing the temporal order of events during granuloma formation in the intestine is challenging. Recently, a novel approach has been published, in which *S. mansoni* eggs are surgically implanted into the sub-serosal tissue of small intestine or colon of mice (29), facilitating the direct and kinetic analysis of egg priming of the immune system in the intestine and within the local draining mesenteric lymph nodes.

### Hepatic granulomas

The formation of granulomas around eggs disseminated into the liver is better understood (Figure 1). In contrast to intestinal granulomas, the liver granuloma cannot be shed and becomes fibrotic over time. Recently, a histological analysis of granulomas revealed that the majority of productive, collagen rich granulomas during natural and experimental *S. mansoni* infection develop in the liver, probably as eggs are trapped in the hepatic tissue as opposed to intestinal granulomas, which appear more organized and with fewer, circumferal collagen fibers (30). Interestingly, hepatic granuloma size decreases from weeks 8 to 20 post-infection, at which point it stabilizes for at least another 32 weeks (10). This down modulation of egg granuloma size reflects the hyporesponsive state of immunity that develops as the infection progresses to chronicity. While normal lobular liver architecture is retained, angiogenesis occurs and contributes both to the genesis of schistosomal portal fibrosis and to fibrotic degradation (31). Indeed, experimentally infected and treated mice showed significant remodeling of hepatic schistosomal lesions over time. A notable feature of the granuloma surrounding the the liver is its protective function to encapsulate hepatotoxic secretions from the egg (26)–in particular omega-1 (ω1) (32).

### The egg-immune interface

It is important to highlight that the egg is a live and biologically active organism that proactively interacts with the host to manipulate immunity and achieve its successful excretion from the host. The shell of the egg itself, aided by factors released from maturing eggs, likely contributes to the initial attachment of the egg to the endothelium. This activating process facilitates granuloma formation and ultimately egg excretion. Indeed, these egg secretions (ES) are the active interface between the egg and the host (33, 34). The egg shell and its ES bear a number of molecules with potential immunomodulatory (IM) activity. The two major secreted egg IM are described in more detail here as they may play an excretion-promoting role in the intestinal granuloma.

#### Alpha-1 (α1)/IPSE/smCKBP

**Figure 2:**
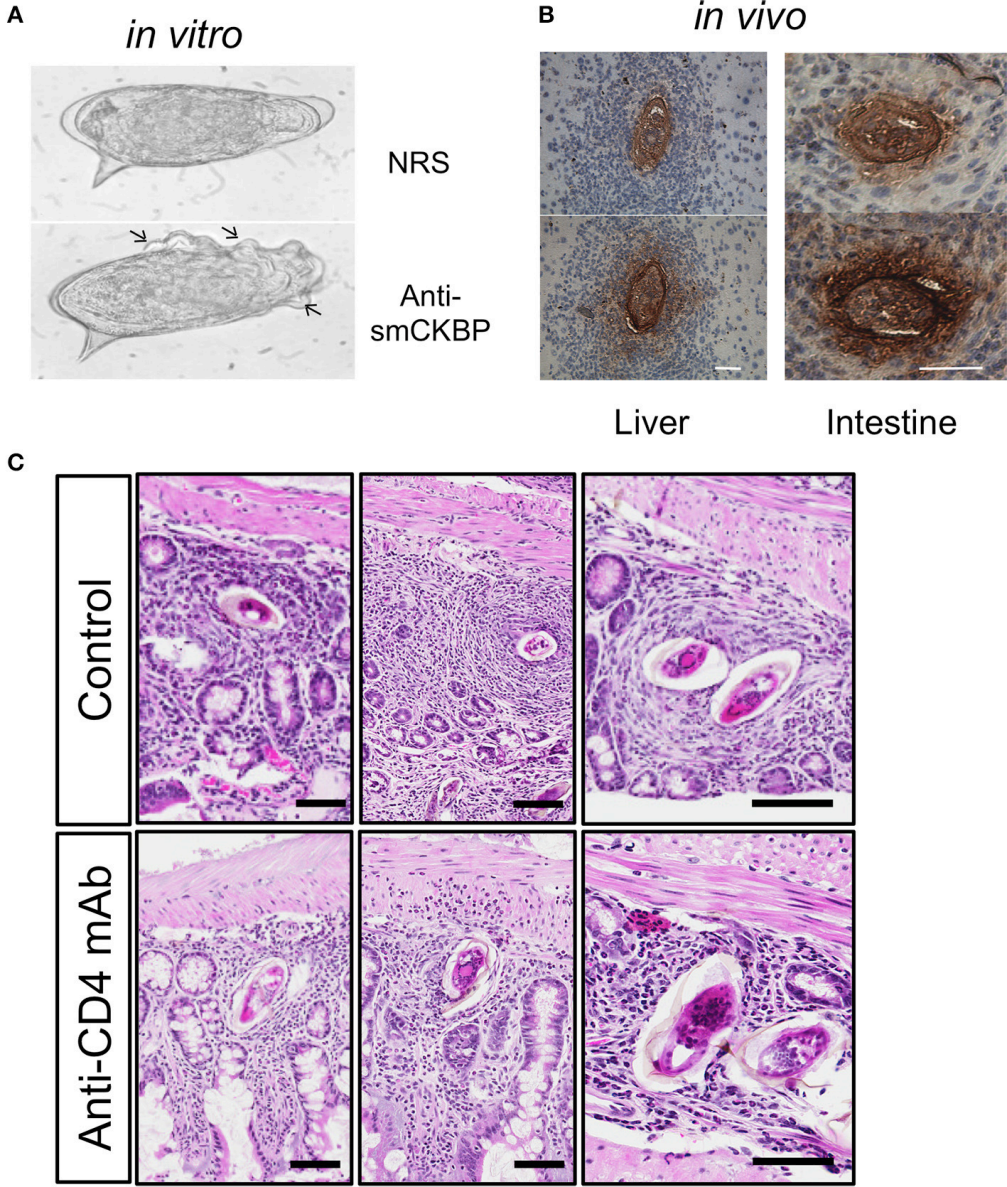
Release of smCKBP from *S.mansoni* live eggs under *in vitro* conditions and detection *in vivo* adjacent to eggs within granulomas in the liver and intestine of infected mice. **(A)** Live eggs were cultured *in vitro* with anti-smCKBP rabbit sera or normal rabbit sera (NRS), with SmCKBP-antibody precipitate formation (arrows) when cultured with anti-smCKBP sera. **(B)** Immohistochemistry detection of smCKBP (brown stain) within the granulomas surrounding eggs in liver or intestines of infected mice. Bar, 50 μm. 2015 Smith et al. Originally published in *The Journal of Experimental Medicine*. https://doi.org/10.1084/jem.20050955. **(C)** Smaller granulomas forming around eggs within intestines of *S. mansoni*-infected mice treated with anti-CD4 mAb compared to control mice, as described (44). H&E-stained sections. Bar, 100 μm.

Alpha-1 (α1) is a dimeric glycoprotein that was first described by Dunne and Doenhoff (35, 36). Haas and colleagues identified that native α1 induced IL-4 release from basophils and cloned the recombinant molecule; subsequently termed IL-4-inducing principle of *S. mansoni* eggs (IPSE) (37, 38). Furthermore, IPSE has a nuclear localization signal in the C-terminus that conveys “infiltrin” activity, an ability to infiltrate through the cell membrane and cytoplasm and translocate to the nucleus (39, 40). Importantly, IPSE/α1 further contributes to the enlargement of hepatic granulomas (41). In many pathogens, host subversion strategies utilize immunomodulators to interact with and manipulate chemokines and alter local cellular recruitment and activation–termed chemokine binding proteins (CKBP) (42). A screen of homogenates or ES from the *S. mansoni* life cycle stages that infect the mammalian hosts identified a CKBP (SmCKBP), within only SEA (~10 μg per mg), that was enriched in ES (~150 mg per mg). SmCKBP selectively bound certain chemokines and when recombinant SmCKBP was administered to mice, it blocked chemokine activity and inflammation (43). SmCKBP has an active role in granuloma formation around live eggs and is released from the maturing egg. This has been evidenced *in vitro* by circumoval precipitation and *in vivo* by detection within hepatic as well as intestinal granulomas (43) (Figures 2A,B). The vastly different biological properties of α1/IPSE/smCKBP–inducing release of IL-4, binding chemokines, infiltrin activity, and enlargement of granulomas–highlight the dynamism and duality of functions for helminth IM. Indeed, the immunomodulating activity of the molecule extends beyond helminths, with recombinant forms shown to suppress inflammation and pathology in models of skin inflammation as well-bladder disease (43, 45).

#### Omega-1 (ω1)

The initial identification of Omega-1, a hepatotoxic egg glycoprotein, is also attributed to Dunne and Doenhoff (32, 36). ω1 possesses both T2 RNase activity (46) and potent Th2 inducing activity (47, 48). The ability of ω1 to prime dendritic cells to elicit Th2 cell expansion is mTOR-independent. Rather, it is dependent on ω1 RNase activity in addition to its native glycosylation, enabling the molecule to bind to the mannose receptor on dendritic cells and be internalized (49, 50). It is noteworthy that both α1 and ω1 within SEA are glycoproteins, with many functional activities of such egg molecules being glycan dependent, requiring interactions with selected C-type lectin receptors on immune cells (51–54).

Recombinant ω1 has been shown to have therapeutic activity in reducing the incidence and development of diabetes in NOD mice (55), modulating inflammasome-dependent IL-1β release (56) and inducing FoxP3 expression in CD4^+^ cell to drive Treg development (57, 58). Regarding metabolic effects, ω1 binds to mouse and human adipocytes and, in mouse models of diet-induced obesity, was shown to induce release of IL-33 resulting in weight loss (59). Further studies are required to investigate whether ω1-induced IL-33-release is responsible for DC modulation and Th2/Treg induction. These immunomodulatory effects may play an important role in regulating optimal granuloma formation and transition through the lamina propria.

## Mechanism of “eggs-cretion”

**Figure 3:**
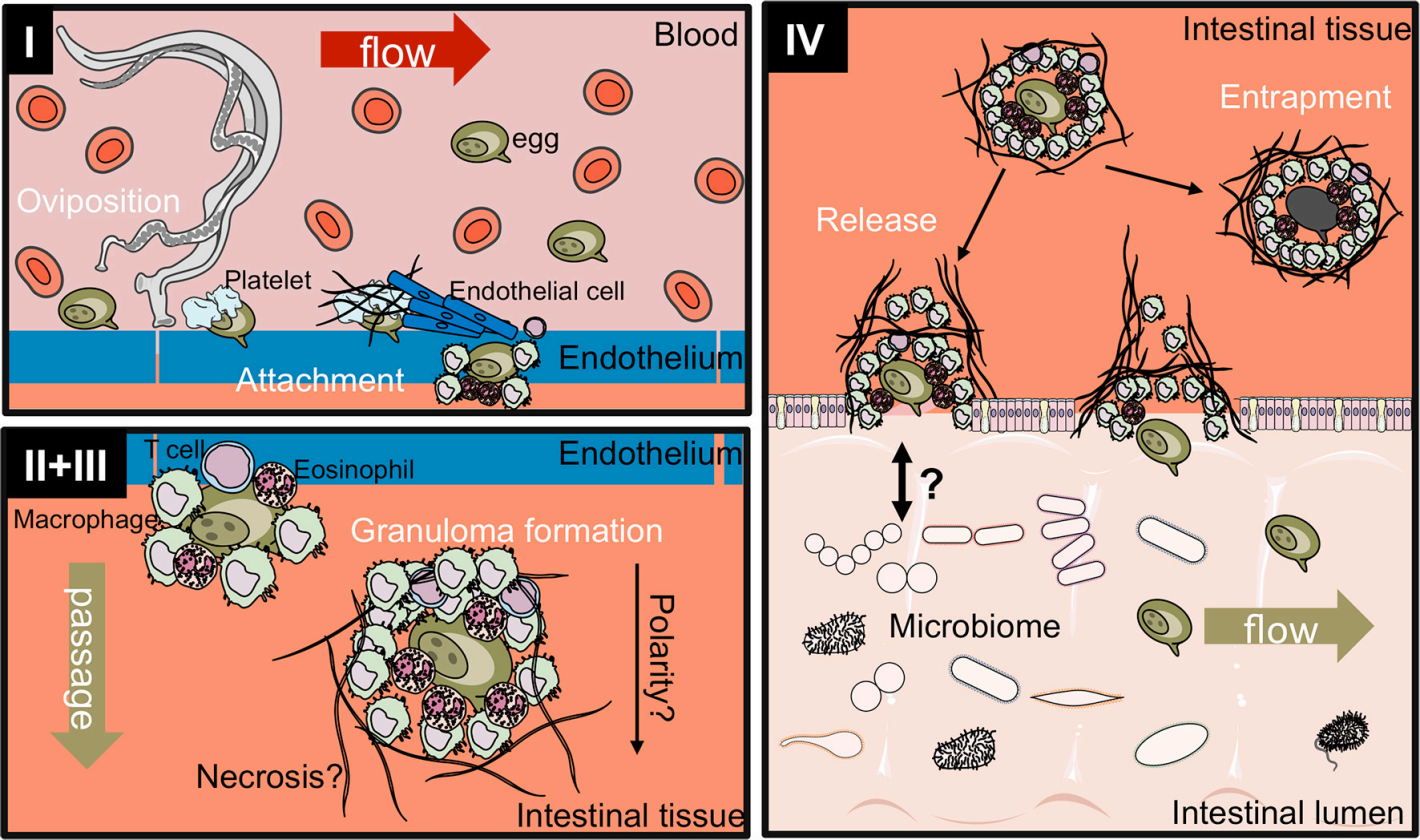
Proposed four-stage process of intestinal egg excretion. I: Adult schistosomes deposit eggs into the vasculature close to the lamina propria. Platelets and fibrinogen adhere to the eggs and activate the endothelium. Endothelial cells actively grow over the egg supporting its extravasation. Eggs that do not cross the endothelial border are disseminated by the blood flow and become trapped mostly in the liver portal system. II + III: Immune cells, such as macrophages, T cells and eosinophils start to encapsulate the egg. Granuloma formation occurs around the egg and together with other processes, such as fibrinolysis, egg secretions-induced necrosis, leads to the passage of the egg toward the intestinal lumen. IV: Entrapped eggs become fibrotic and calcified. Interaction with the microbiome, epithelial cell death and remodeling may lead to the active release of eggs, which are then released to the environment with host feces. Illustrations modified from Servier Medical Art, licensed under a Creative Common Attribution 3.0 Unported License.

While the *S. mansoni* egg excretion process still remains ill-defined to date, it can be broadly divided into four stages (Figure 3):

- Egg release into the bloodstream and attachment to the endothelium
- Immune-dependent granuloma formation
- Transition between endothelium and epithelium
- Release into the intestinal lumen

### Stage I. egg release into the bloodstream and attachment to the endothelium is triggered by adult worm and egg secretions

Within the mesenteric vasculature, adult male and female schistosomes reside. Here, they move against the blood toward the endothelium facing the intestinal epithelium as a response to a nutrient gradient. Female *S. mansoni* worms release roughly 300 eggs per day–equalling approx. one egg every 5 min–a rate that stays constant for at least 1 year (10). Although female worms flex backwards during oviposition (thereby releasing the egg in close proximity to the endothelium), active penetration of eggs through the endothelium is unlikely (34). The metabolic enzymes enolase (phosphopyruvate hydratase) and glyceraldehyde-3-P-dehydrogenase (GAPDH) have been identified as parts of the eggshell and act as surface receptors to bind plasminogen (60, 61) increasing the fibrinolytic activity of the egg. Further, lack of enolase has been shown to decrease binding of other pathogens to the endothelium (62, 63). Although both enolase and GAPDH are present in exosome-like vesicles (ELVs) produced by adult *S. mansoni* worms (61), it is not known if ELVs are also released from the egg stage. The release of such metabolic enzymes within the surrounding egg milieu induces a localized metabolic niche that modulates local cell functions to achieve egg excretion.

Indeed, human enolase has been shown to activate pulmonary endothelial cells and to increase surface expression of the cellular adhesion molecule ICAM-1 (64). It has been reported that endothelial cells actively migrate over freshly deposited eggs *in vitro* (65), a process enhanced in the presence of sera. Furthermore, eggs can bind to and activate platelets, which may subsequently activate endothelial cells (66). This process also seems to contribute to extravasation, with thrombocytopenia impairing egg excretion (67). Interestingly, schistosome eggs also bind other host plasma proteins, including von-Willebrand factor, which may assist in the initial attachment to the damaged or activated endothelium (68). Enolase, present in the egg shell, can also act as a plasminogen receptor and induce plasminogen activation and plasmin generation (69). Plasmin can subsequently contribute to blood clot lysis (important for adult worms) and may induce monocyte recruitment (70).

The initial recruitment of immune cells to the site of granuloma formation is dependent on cellular adhesion molecules, such as ICAM-1. ICAM-1 is upregulated by SEA and high expression can be observed within hepatic granulomas (71). Here, it is essential for granuloma formation, especially during early acute stages (72–74). Ileal and colonic granulomas also showed high expression of ICAM-1 as well as LFA-1 and VLA-4 in acute and chronic infection (75). In ICAM-1-deficient mice, VCAM-1 was upregulated in hepatic granulomas, whereas no expression of ICAM-2 or PeCAM was observed (71). Interestingly, close correlation has been identified between soluble ICAM-1 and fecal egg counts from infected patients feces (73) where it was proposed to function as a negative regulator by inhibiting leukocyte recruitment and downmodulation of granuloma formation.

It has been suggested that the presence of the characteristic lateral spine of the eggs may facilitate attachment to the endothelium, perhaps causing cell damage to elicit a danger signal and while intestinal peristalsis may also contribute to the extravasation process, its actual function remains unclear. However, a study published in 2013 showed that the location of egg deposition within the intestinal tract may contribute to efficient egress of eggs (76). *S. mansoni* eggs are significantly more abundant in areas with Peyer's patches, where egg secretions cause loss of cellularity facilitating transition. Indeed, in support of this, mice without Peyer's patches excrete fewer eggs (76).

### Stage II. immune-dependent granuloma formation is driving egg excretion

The main mechanism that facilitates egg excretion is the formation of the granuloma around the egg. As most findings concerning granulomatous inflammation have been found in hepatic granulomas, the following events have to be treated with caution as experimental data for the formation of intestinal granulomas is largely missing.

It has been noted earlier that the process of *S. mansoni* egg excretion is an exquisite, immune-dependent process (Figure 3) as illustrated by significant reduction in fecal egg excretion in T cell- (44, 77, 78) and nude mice (79). Severe combined immunodeficient mice were almost incapable of passing parasite eggs in the first weeks of oviposition (80, 81). Indeed, *S. mansoni*-infected HIV+ patients with acquired immunodeficiency syndrome had fewer eggs in their feces than HIV seronegative patients with the same levels of *S. mansoni* infection (82, 83), which correlated with CD4 T cell counts after anti-retroviral therapy (84). However, the role for CD4^+^ cells in schistosome egg excretion of humans was not evident in studies on other HIV+ patient cohorts (85, 86).

Strikingly, mice deficient in the Th2-associated cytokines IL-4 and IL-13 passed almost no eggs in their feces (23). IL-4-deficient mice display significantly impaired granuloma formation and increased mortality associated with intestinal pathology (22). This is also observed in mice with a combined deficiency in IL-4 and IL-13 (23) which also develop fatal endotoxemia. Furthermore, IL-4-deficient mice show a significant accumulation of eggs in the intestinal wall, a finding supportive of the function of the granuloma in facilitating egg excretion (23). Similarly, mice with specific deficiency in IL-4 and IL-13 within T cells succumb to acute *S. mansoni* infection (25). Also, IL-4Rα^−/−^ mice may develop fatal hemorrhaging following *S. mansoni* indicative of a protective role of the granuloma from exacerbated immunopathology in the intestine (87). IL-4Rα-signaling involves both IL-4- and IL-13-mediated receptor engagement and the particular role of IL-13 was revealed to drive hepatic fibrosis (23, 88).

While there has been a focus on T cells, other immune cells also contribute to both granuloma formation and the excretion of eggs. Indeed, eosinophils are a major constituent of intestinal granulomas (89). Although their precise role remains unclear, it has been reported that eosinophils may promote egg excretion (90). However, studies using anti-IL-5 antibody-mediated eosinophil blockade found normal numbers of eggs passed in the feces (91, 92), with no marked alterations in hepatic granuloma formation noted in transgenic mouse models of eosinophil deficiency (93). Another possible function of granuloma eosinophils may be the destruction of miracidia within the surrounded (trapped) egg (94), in part explaining the higher abundance of eosinophils in hepatic granulomas (28, 30) and the occurrence of eosinophil pyroptosis *in vivo* (95).

Basophils can be directly activated by IPSE/α1 to release IL-4 (38) and are present in intestinal granulomas (96). Additionally, a new molecular mechanism of action has been revealed whereby IPSE, a member of the βγ-crystallin superfamily, can bind IgE through the IPSEs crystallin fold thereby activating basophils independently of formal IgE cross-linking (97). Although basophils have been shown to drive Th2 polarization in other helminth infection models (98, 99), whether or not they contribute to intestinal granuloma formation and egg excretion is unclear to this date (25, 100).

While neutrophils are found to acquire a pro-inflammatory phenotype during *S. japonicum* infection (101), they do not seem to participate in the granuloma formation in *S. mansoni* infection (87), which may be in part due the presence of the kunitz-type protease inhibitor SmKI-1, capable of inhibiting neutrophil function (102).

The more recently described group of innate lymphoid cells (ILC) have gained a lot of attention as they are capable of initiating type 2 immune responses against certain helminths and during allergic responses (group 2), maintaining intestinal homeostasis (group 3), and enhancing type 1 responses via IFN-γ release [group 1; reviewed in (103, 104)]. To date, only limited data is available on the contribution of ILC, and in particular ILC2, during *S. mansoni* infection toward granuloma formation and egg excretion. In a pulmonary model of schistosome egg injection we have shown that ILC2 can contribute to pulmonary fibrosis (105). Because ILC2 are abundant in the intestine and they can directly interact with CD4 T cells to enhance polarization toward Th2, they may play non-redundant roles in the excretion process by instructing T cell responses. ILC2 are mainly activated by the alarmin cytokines IL-33, IL-25, and TSLP to produce IL-5 and IL-13. While individual ablation of each of these cytokines did not impair hepatic granuloma formation in chronic schistosomiasis, the combinatorial targeting of all three cytokines led to reduced hepatic fibrosis, impaired eosinophil recruitment and fewer numbers of IL-13-producing ILC2 (106). Thus, while the impact on early intestinal granuloma formation and egg excretion remains obscure, the marked effect of alarmins is highly likely to impact on intestinal granuloma formation. Omega-1 released from eggs within the intestines may–in addition to cytotoxic effects–increase the release of IL-33 and therefore the activation of ILC2. Additionally, ILC3 might exert similar functions as they have been shown to be involved in the maintenance of tolerance toward commensal microbiota through the interaction with CD4 T cells in the gut (107). Interestingly, altered numbers of ILC2 have been detected in the circulation of *S. haematobium*-infected patients (108). However, further investigation is required to fully elucidate the role of ILC populations in schistosoma infection and granulomatous inflammation.

Dendritic cells (DCs) are capable of processing and presenting schistosome egg antigens (109) and depletion of DCs during an ongoing *S. mansoni* infection severely disrupts the generation of the Th2-polarized immune response (110). While hepatic conventional DCs act in an immunogenic, rather than tolerogenic, capacity, their function in the intestine may be different (111). Using an injection model for the direct delivery of schistosome eggs into the subserosal intestinal tissue revealed that IRF4-expressing CD11b^+^ DCs promote Th2 responses in the intestine (29). It further has been shown that Omega-1 is the major component conditioning DCs for Th2 polarization (48).

Macrophages are the most abundant cell population in intestinal granulomas (28). During *S. mansoni* infection of immunocompetent mice macrophages acquire a protective alternatively activated phenotype through IL-4- and IL-13-mediated IL-4Rα-signaling. The M2 phenotype is associated with the increased expression of Arginase-1, an enzyme converting L-arginine to L-orthinine, which is further converted to proline–a critical amino acid for the production of collagen, and therefore, the development of fibrosis (112). While this process is detrimental to host survival through fibrotic liver damage, it might not be as critical in the intestinal granulomatous response as the surrounding granulomas are overall more temporarily restricted. Therefore, other properties of IL-4Rα-mediated M2-polarized macrophages, such as IL-10 production, PD-L2 and Relmα expression, may be more crucial to prevent aberrant inflammation.

When macrophages are unable to respond to IL-4 and IL-13 cytokine signals in IL4RαLysMCre mice the hosts succumb to endotoxemia, which can be in part rescued by antibiotic treatment (87). More inflammatory cells were observed surrounding intestinal granulomas suggesting that M2 macrophages are specifically required to prevent excessive damage to the intestinal wall and promote the efficient transport of eggs into the intestinal lumen. In contrast, Arginase-1-expressing macrophages were shown to limit Th2 responses (113) and prevent the formation of exacerbated granulomas in the liver. Later, it was found that Lyz2lo macrophages were able to escape LysMCre-mediated IL-4Rα-deletion and acquire M2 properties in response to *S. mansoni* egg injection, which may account for some of the observed differences between studies (114). It was further shown that during *S. mansoni* infection macrophages require IL-4/IL-13 released from Th2 cells to acquire an M2 phenotype (25). Importantly, granuloma formation was significantly impaired in mice with T cell-derived IL-4/IL-13-deficiency and egg excretion in the feces was compromised, although it did not reach statistical significance. These results suggest that while M2 macrophages contribute to the increased fibrosis surrounding the trapped eggs in the liver, they also promote efficient shielding of the eggs in their transition through the intestinal tissue in order to prevent excessive damage. Macrophage phenotype and granuloma formation is ultimately determined by Th cell polarization.

T cells are the most important cells for successful egg excretion in the intestine. Antibody-mediated depletion of T cells was shown decades ago to lead to impaired granuloma formation, egg retention and exacerbated disease resulting in increased mortality (78). Later targeting of L3T4 (CD4) T cells phenocopied anti-CD3 mediated depletion highlighting the role of the T helper subset (115). In *S. mansoni* infected mice subjected to anti-CD4 mAb-depletion the size of the granuloma is significantly reduced in liver and intestines [Figure 2C, (116)]. Furthermore, the cellular granuloma composition in CD4-depleted mice consists of fewer eosinophils and more neutrophils, which are virtually absent in the eosinophil-rich granulomas of immunocompetent mice. Similarly, the absence of T cell-derived IL-4/IL-13, e.g., the functional absence of Th2 cells, impairs granuloma formation and egg excretion, and was found to increase mortality (25).

The critical role of Th2 cells for the protection of host tissue from cytotoxic effects and correct granuloma formation is further substantiated by studies showing that injection of the Th1 cytokine IFNγ interferes with granuloma formation and IL-12- deficiency–in fact Th1-ablation–increases granuloma size and Th2-mediated inflammation (117, 118). Although Th1 cells seem to be required for the early release of IFNγ and IL-2 facilitating granuloma formation as part of a delayed type hypersensitivity response, prolongation of the Th1 response led to increased pathology and mortality (44, 117, 119, 120). Furthermore, it was shown that hepatosplenic schistosomiasis in human patients is associated with increased levels of TNFα and IFNγ, while type 2 cytokines are reduced (121).

IL-17-producing Th17 cells have also been implicated in schistosome granuloma formation as IL-17 levels in susceptible and resistant mouse strains correlated with granuloma formation (122). This has been shown to be a direct effect on the IL-23 and IL-1 release of DCs in response to SEA (123, 124). In the context of the roles of egg glycans in immune priming, theses pathological effects are mediated through the C-type lectin receptor CD209a (125).

In the absence of Th1 cells (*Tbet*^−/−^) Th17 cells drive exaggerated inflammation with an increase of neutrophil infiltration into the granuloma, while the combined absence of Th1 and Th17 cells led to smaller granulomas with increased type 2 biased infiltration of M2 macrophages and eosinophils (126).

Regulatory T cells expand during the chronic stage of *S. mansoni* murine infection and are present in *S. mansoni* infected patients (127–129). It has speculated that immunosuppression by Treg cell by the release of IL-10 and TGFβ may limit tissue pathology toward trapped eggs during infection (130–132).

The role of B cells and antibodies in *S. mansoni* infection of mice has been extensively investigated. While B cells were found to be involved in the development of Th2 polarization, their role in granuloma formation is still not entirely clear (133–135). Antibodies specific for SEA arise after egg deposition and expand throughout infection (136) and multiple studies have shown regulatory roles of antibodies in granuloma formation (137–139). Interestingly, immune complexes of chronically infected patients were capable to inhibit granuloma formation *in vitro* (140). Indeed, infection of Fc-receptor deficient mice (γ or ε) led to the formation of larger and collagen-rich granulomas (138, 139), suggesting regulatory potential of Fc-bearing effector cells within the granuloma or sequestration of egg antigens. Whether this process is involved in the maintenance of intestinal granulomas remains to be investigated.

Identification of regulatory B (Breg) cells in response to schistosome eggs (141) and their production of IL-10 in response to the schistosome glycan LNFPIII (142, 143) led to subsequent studies using live *S. mansoni* infections (144–146). Indeed, α1 drives the expansion of Breg cells (147). Because Bregs arise late during chronic infection it seems unlikely that they contribute to the natural intestinal granuloma formation facilitating egg excretion and rather may contribute to limit chronic intestinal inflammation.

The functions of the tuft cell as IL-4Rα-expressing IL-13-responsive cell type in the intestine has been described during gastrointestinal nematode infections (148–150). Whether these cells contribute to the intestinal granuloma formation by their IL-25 release or facilitate the egg excretion process during *S. mansoni* infection has not been investigated.

Taken together, egg excretion through the intestinal wall can only be achieved in the presence of a Th2-biased granulomatous inflammation. All cells described here take part in the shaping of the intestinal granuloma and may therefore influence Th2 polarization, toxicity protection and promote or inhibit egg excretion. While the roles of CD4^+^ T cells and macrophages is well-understood, more research is required focusing on other immune cells function in intestinal granulomas.

Yet, the question remains: What drives the egg+granuloma to transit from the endothelium toward the epithelium?

### Stage III. transition through the lamina propria is achieved by toxic effects and displacement

The mechanism of how the viable egg transits through the intestinal lamina remains elusive, however, some findings suggest a role for the developing granuloma, extracellular matrix degradation, and schistosome egg antigens.

Recently, the collagen, fibronectin and plasmin contents of intestinal granulomas have been investigated (151). After extravasation, fibrinolysis can be observed around the biologically successful eggs, i.e., those that will achieve egg excretion, in contrast to trapped eggs that are not excreted (Figure 3). Plasma fibronectin is part of the blood clotting and wound healing response and it was recently found that *S. mansoni* is able to express extracellular tegumental calpains that cleave fibronectin (152). Fibronectin was also found in early granulomas, while chronic granulomas are collagen-rich (30, 153). Eggs also express SmCalp1 and may actively contribute to the fibronectin degradation (152). Further, SmEnolase, is highly expressed by eggs, and is able to promote plasminogen activation (69). Plasmin degrades fibrin and fibronectin–among other targets–and thereby contributes to the degradation of extracellular matrix proteins. Indeed, a striking difference exists between hepatic and intestinal granulomas with regard to perioval fibrin and fibronectin deposition (151). While they were found in 23 and 77% in hepatic granulomas, respectively, only 3 and 11% were positive in the intestine. Viable eggs actively degrade fibrin and fibronectin, therefore the increase of these ECM components in hepatic eggs might stem from the increased proportion of dead eggs. However, as the data set is limited, in-depth investigation of the plasmin, fibrin, fibronection, and collagen content of intestinal granulomas is warranted. Fibrinolysis and plasmin around eggs could be a result of macrophage invasion or proteolytic activity of the eggs. At the moment, it remains unclear what the exact order of events in fibrin and collagen deposition, plasminogen activation and fibrinolysis is and thus a more detailed investigation is required.

While collagen deposition leads to immunopathology in the liver, collagen degradation may be more important in intestinal granulomas. Eight weeks after infection the collagen content in hepatic granulomas is already significantly increased but only minimal deposition is observed in the small intestine and even less in the colon (154). While collagen content increased over time in all three organs, in intestinal granulomas collagen marks chronic granulomas–and therefore trapped eggs (153). Furthermore, collagen structure appeared concentric in the liver while a discontinuous deposition was observed in the intestine (154). While eggs themselves were not able to degrade collagen *in vitro* (155), egg-activated plasmin may contribute to collagen degradation. Furthermore, M2-like macrophages can degrade collagen in a mannose receptor-dependent manner (156). Additionlly, many matrix metalloproteinases are expressed during *S. mansoni* infection with elevated expression of MMP-2, MMP-3, and MMP-8 transcripts in the colon of chronically infected mice (157), all of which possess collagenase activity.

While this array of ECM-degrading mechanisms may promote the movement through the lamina propria–but where does directionality come from? One possibility is that the influx of immune cells follows the egg through the initial crossing of the endothelium. Therefore, the granuloma first develops basally at the egg. The first cells may shield from the cytotoxic effects of the SEA, while SmEnolase and calpains exert their effects apically and degrade the ECM in front of the egg. Over time, macrophages will surround the egg and become activated by IL-4 and SEA to promote collagen degradation. It was shown that excretion is a relatively quick process compared to the development of liver fibrosis (158), therefore collagen deposition may occur only at the rim or basally of the granuloma to maintain the surrounding tissue architecture, while the apical ECM is degraded and more cells and fluid infiltrates from the basal side displacing the egg forward toward the epithelium. Simultaneously, cells around the egg succumb to the cytotoxic effects of IPSE/α1 and ω1. If the cells infiltrate from the basal side of the granuloma the cells at the apical side are more exposed to the cytotoxic molecules and therefore the first to die from necrosis. Further studies using intravital microscopy approaches are required to get more insight into the mechanisms by which the granulomas move the egg through the intestinal wall.

Another possibility is that because eggs are more or less deposited at the same site in the vasculature and often more than one egg is found in a granuloma, the intestinal tissue may develop an ill-defined “tunnel” structure with more collagen, fibrin and fibronectin deposition at the “tunnel wall,” while the inner tissue has a less rigid composition from repeated ECM degradation. Peristaltic movement of the bowel may also play a supportive role in moving the egg+granuloma along the beaten path. However, to date there is no experimental data available to support this theory, mainly because the larger granulomas surrounding unsuccessful eggs trapped in the intestinal wall may gain more attention and are more similar to what we expect from the research on hepatic granulomas than smaller successful egg granulomas.

### Stage IV. release into the intestinal lumen

The process of actual release of the egg after transit through the intestinal wall into the gastrointestinal lumen has not–to our knowledge–been addressed in detail experimentally. The parasite eggs that are found in the feces of infected humans, as well as experimental mice, are devoid of an encapsulating granuloma suggesting that the egg leaves the granuloma as it enters the fecal matter. This poses the questions: How does it leave the granuloma? What happens to the remaining granulomatous tissue in the intestinal epithelia? A possible mechanism of granuloma resolution may be interaction of microbiota and the immune cells at the outer rim of the granuloma that come in direct contact with the mucous layer and get successively destroyed concomitant to the cytotoxic and fibrinolytic effects mediated by the egg and its secretions. Whether the composition of the mucus layer affects the survival of schistosome eggs is currently unclear. After the egg is released the remaining cells in the granuloma will likely be resolved as part of the intestinal wound healing response, where the already present macrophages may contribute to (Figure 3). Clinically, the colonic mucosa becomes atrophic and acquires a granular yellowish appearance (159), which are probably macrophages from the granulomas shed with tissue remodeling.

One complication observed in patients is the occurrence of colon polyps that are shed and cause intestinal bleeding (160). Probably this may happen when unsuccessful trapped eggs become calcified, losing their fibrinolytic potential and leading to chronic granulomas as seen in the liver.

#### Microbiome influences on egg excretion

In recent years it became increasingly understood that the microbiome composition will impact on the outcome of most immune responses (161) and perturbations of this complex system may lead to detrimental consequences for the hosts health (162–164). A significant alteration of the microbial communities that impacts on the hosts immune response has been shown for gastrointestinal helminth infections with *Heligmosomoides polygyrus* (165), *Trichuris muris* (166), and *Trichinella spiralis* (167).

With respect to *S. mansoni* infection one study found that the absence of gut microbiota through the administration of antibiotics alters the immune response against *S. mansoni* (168). Further, intestinal inflammation was significantly reduced in antibiotics treated mice and fecal egg secretion was impaired. As the microbial translocation in immunocompromised mice will only occur after the first eggs have traversed the intestine–or at least entered it to compromise the integrity of the gut barrier–the importance of the microbiome composition for egg secretion itself becomes evident. In infected humans, while one study failed to find statistically significant differences (albeit a relatively small cohort size) between the microbiome composition of non-infected, *S. mansoni*-infected and praziquantel-treated children (169), it has to be noted that another study found significant differences in *S. heamatobium-*infected children (170).

For future studies it would be interesting to infect germ free or gnotobiotic mice to further investigate the impact of the microbiome during schistosome infection on the immune response or to comprehensively analyze the microbiome composition of infected mice and perform fecal transplantation experiments.

## Conclusion and future directions

Research over the past decades has uncovered many fascinating facets of *Schistosoma mansoni* biology and the host immune response against both worms and eggs. The immunology of egg granuloma formation has been extensively investigated due to its central role in *S. mansoni* infection-associated immunopathologies. We previously posited on helminth immunobiology that despite such discoveries, there are many *unknown unknowns* yet to be not only identified but also to be experimentally investigated (171). Indeed, the processes involved in the granulomas evolutionary role in facilitating egg transit through the intestinal wall is still relatively unclear. With more sophisticated approaches, lessons learned from studying the hepatic granulomas, and ever more transgenic mouse models together with translational studies, the mechanism of egg transit may finally be unveiled.

Novel techniques, such as the injection directly into the epithelium will greatly improve our understanding of the intimate processes happening in the formation of a functional egg granuloma within the intestine (29). However, the initial interaction of the egg with the immune system–when it is deposited in the blood stream–will not be accessible by this system. High-resolution ultrasonography has been recently shown to be applicable for noninvasive time-course observations of hepatic lesions in *S. japonicum* infection in mice (172). Furthermore, the development of other noninvasive techniques, such as small-animal PET-MRI or intravital 2-photon microscopy may be helpful tools to study early events of granuloma formation, as undertaken in the liver (173). Furthermore, the manipulation of *S. mansoni* eggs by lentiviral transduction, as it has been shown for ω-1 knockdown (174), will make noninvasive observation techniques more powerful. Identification of immune-modulation egg products has also been facilitated by the fact that the cost and availability of core facilities for transcriptome sequencing became feasible (175) and “omics” data is made available at WormBase ParaSite (176). Using the lentiviral knockdown model or schistosome egg-antigen coated beads (177, 178) either *in vivo* or in *in vitro* models, such as intestine-on-a-chip (179), intestinal organoid cultures (180), or other novel models (181, 182), are potential approaches to provide new insights.

Future studies on this unique *S. mansoni* immune dependent “eggs-cretion” process will reveal novel insights on the host-pathogen-interface that will impact on our understanding of fundamental functions of the immune system in both health and disease.

## Author contributions

Both authors have made substantial, direct and intellectual contributions to the work, and approved it for publication.

### Conflict of interest statement

The authors declare that the research was conducted in the absence of any commercial or financial relationships that could be construed as a potential conflict of interest.
